# Food Pathways of *Salmonella* and Its Ability to Cause Gastroenteritis in North Africa

**DOI:** 10.3390/foods14020253

**Published:** 2025-01-15

**Authors:** Mohamed-Yousif Ibrahim Mohamed, Hazim O. Khalifa, Ihab Habib

**Affiliations:** 1 Department of Veterinary Medicine, College of Agriculture and Veterinary Medicine, United Arab Emirates University, Al Ain P.O. Box 1555, United Arab Emirates; hazimkhalifa@uaeu.ac.ae; 2 ASPIRE Research Institute for Food Security in the Drylands (ARIFSID), United Arab Emirates University, Al Ain P.O. Box 1555, United Arab Emirates

**Keywords:** *Salmonella*, food chain, virulence genes, antimicrobial resistance, North African countries

## Abstract

Infections caused by human pathogenic bacteria in food sources pose significant and widespread concerns, leading to substantial economic losses and adverse impacts on public health. This review seeks to shed light on the recent literature addressing the prevalence of *Salmonella* in the food supply chains of North African countries. Additionally, it aims to provide an overview of the available information regarding health-related concerns, such as virulence genes, and the presence of antibiotic resistance in *Salmonella*. This review highlights a gap in our comprehensive understanding of *Salmonella* prevalence in the food supply chains of North African nations, with limited molecular characterization efforts to identify its sources. Studies at the molecular level across the region have shown the diversity of *Salmonella* strains and their virulence profiles, thus, these results show the difficulty of controlling *Salmonella* infections in the region. In addition, the discussion of antibiotic resistance makes it clear that there is a need for the development of comprehensive strategies to fight the potential threat of antimicrobial resistance in *Salmonella* strains. Despite common reports on animal-derived foods in this region, this review underscores the persistent challenges that *Salmonella* may pose to food safety and public health in North African countries.

## 1. Introduction

*Salmonella* continues to pose a significant threat to public health worldwide, contributing to approximately 93.8 million cases of foodborne illness annually and imposing a substantial economic burden. This burden includes an estimated 150,000 deaths each year [1,2]. In the North African area, numerous foodborne diseases are prevalent and the food safety systems are confronted with different difficulties. As a result, determining the factors influencing *Salmonella* contamination along the food chain in the North African region should be a priority to put in place effective prevention and control measures [3]. The contamination of food and the environment by *Salmonella* is very common in many samples across North Africa; hence, the people of this region are at the risk of public health hazards [4,5]. The outbreaks of the illnesses caused by *Salmonella* show mortality and hospitalization, hence, the economic losses. Limited resources and regulatory challenges hinder comprehensive control efforts, emphasizing the significance of *Salmonella* as a concern in the region [6].

The North African region, which includes Algeria, Egypt, Libya, Morocco, and Tunisia, is in a situation of having to face the problem of the scarcity of water, which is the main issue of this region. On the whole, the area is made up of around 4,758,160 square kilometers, 90% of which is desert or semi-desert, which are the regions that are responsible for food infection prevention and, in this setting, *Salmonella* is the most common cause of foodborne diseases; therefore, it is responsible for the majority of the foodborne illnesses. The high incidence of *Salmonella* in food [7] and environmental samples [8] shows that this bacterium still exists in the whole food chain in the region [9].

Numerous factors are behind the problems associated with the control of *Salmonella* in the North African food chain. Negative infrastructure for food production, processing, and distribution, along with poor hygiene practices, lack of sanitation, and no access to clean water, create suitable conditions for *Salmonella* contamination [9]. *Salmonella* infections can make the body more resistant to the pathogen and, thus, may decrease hospitalization and deaths [10]. Resistance can be caused by the immune system which is activated by the previous exposure to *Salmonella* [11,12]. Hence, individuals with the acquired resistance may have the milder symptoms or even the asymptomatic infections, which in turn, will cause a reduction in hospitalization and death. However, even if resistance can be partial in the lowering of the *Salmonella*-related illnesses, in some cases, it does not eliminate the risk entirely [10]. The absence of robust surveillance systems and accurate data on hospitalization and death rates in North Africa poses a big problem in obtaining the exact picture of health issues such as *Salmonella* infections. Without comprehensive data, drawing conclusive conclusions about the prevalence and impact of *Salmonella* becomes difficult [7]. Nevertheless, indirect evidence such as outbreak reports, case records in healthcare facilities, and food contamination studies is another way of providing the link to the problem [13,14,15].

*Salmonella*, a Gram-negative bacterium from the Enterobacteriaceae family, is a primary cause of gastroenteritis in developed countries, with *Salmonella enterica* being the most frequently mentioned [16]. The prevalence of salmonellosis raises significant public health concerns in various European countries [17]. The ability of *Salmonella* to survive and replicate within human macrophages is a key factor in the pathogen’s virulence and its resistance to treatment. This intracellular survival strategy complicates both the diagnosis and management of *Salmonella* infections, making it a major challenge for public health. Efforts to combat *Salmonella* infections need to account for its ability to persist within immune cells and develop novel therapeutic strategies to target these hidden reservoirs of infection.

Animal or animal product specialists, for instance, veterinarians, farmers, butchers, slaughterhouse workers, and cooks, are the ones who are at higher risk of being exposed to *Salmonella* through their occupational activities. Uniquely, the *Salmonella* that is introduced into the human body can sometimes make the body resistant to the pathogen, just like a kind of immunity [18]. Nevertheless, there is a negative aspect to this situation as well. Although these persons may acquire resistance, they also become the carriers of *Salmonella* themselves, like Typhoid Mary and, hence, they can spread the disease to others. In other words, they can carry the bacteria without having any symptoms of the disease and, thus, they could be spreading it to others [10]. The results of such transmission are different, depending on factors like the health and immunity of the receivers and the particular strain of *Salmonella*. Hence, although occupational exposure may bring a certain degree of immunity, on the other hand, it also emphasizes the need for strict hygienic measures and preventive measures to reduce the infection risk and guarantee public health safety [19]. Human salmonellosis generally presents itself as a slight to moderate, self-limiting gastrointestinal infection with symptoms like abdominal pain, diarrhea, vomiting, cramps, fever, and nausea. However, severe cases leading to fatalities can occur [17]. In case of recurrent infection, *Salmonella enterica* may invade the parts of the body other than the intestinal tract, making local infections or invasive infections more likely, especially in people with compromised immune systems, the elderly, or children.

The emphasis on the importance of infection prevention is crucial because the research on salmonellosis in humans in Africa is minimal as far as North African countries are concerned. The problem of the knowledge gap is important to solve as soon as possible because *Salmonella enterica* infections are expected to result in 3.4 million human illnesses, with Africa having the highest burden of the disease [20].

This review aims to: (i) provide background knowledge and (ii) highlight recent advancements on the occurrence of *Salmonella* and its virulence genes among serotypes in the food supply of North African countries, based on studies published over the past 20 years. As a secondary objective, we also aim to shed light on the existing data regarding the present trend of antimicrobial resistance (AMR).

## 2. Study Design and Methodology

An extensive literature search of electronic databases, including PubMed (https://pubmed.ncbi.nlm.nih.gov/) and Google Scholar (https://scholar.google.com/), was conducted to identify relevant studies published over the last two decades [21]. To ensure the reliability and scientific rigor of the information presented, we excluded non-peer-reviewed sources, including letters to the editor, opinion pieces, and anecdotal reports. Three keywords for each country were employed to refine the search, such as “*Salmonella* in food Algeria”, “*Salmonella* virulence genes Algeria”, and “Antimicrobial resistance *Salmonella* Algeria”.

**Figure 1:**
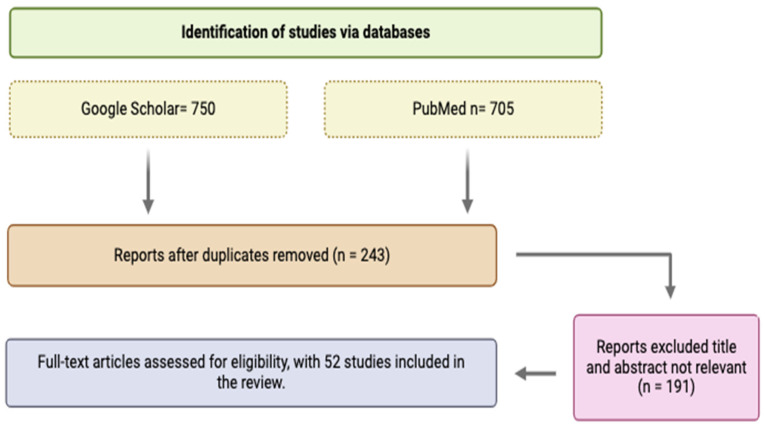
PRISMA 2020 flow diagram for updated reviews in the database search. Created using Biorender.com (accessed on 15 June 2024).

The initial search yielded a large number of articles (Figure 1) which were then subjected to a rigorous screening process. The inclusion criteria included studies that reported on the *Salmonella* prevalence, serotypes, antimicrobial resistance patterns, and the virulence gene profiles of the samples from North African countries. The studies that were not found to fulfill these criteria or that did not have enough data were dropped from the review [22]. Figure 1 represents the systematic approach to the inclusion and exclusion of articles.

## 3. Current Status of Human Salmonellosis in North Africa

The current status of human salmonellosis in North Africa shows the complex relationship between the prevalence of *Salmonella* infections and various factors, especially its link to food [7]. Contemporary researchers have turned their attention to the extremely high contribution of food-related sources in transmitting *Salmonella* to humans in the region. Meat products especially have become major factors in salmonellosis cases, posing a threat to the health of the nation [23,24,25]. The epidemiological landscape in North Africa is affected by different elements such as the population, healthcare system, and environmental factors. Infectious diseases like *Salmonella* have a great impact on the region, and their occurrence is influenced by localized practices and resources [7]. Despite the difficulties, there is now an increasing awareness of the necessity of the data-driven approach for monitoring and control which will be effective for infectious diseases. The cooperation between health authorities and institutions is essential for the protection and ensuring of public health in North Africa [19].

The epidemiological landscape shows different prevalence rates in North African countries [5,26,27,28,29] which, therefore, leads to the emphasis on the need for a detailed understanding of the dynamics involved [7]. Elements such as cultural customs, food handling, and processing techniques significantly affect the occurrence of *Salmonella* infections. Additionally, the increasing globalization of food supply chains has introduced new challenges in managing and controlling the spread of these pathogens [30].

In a study conducted in Algeria by Djeffal et al. [23], it was found that among human *Salmonella* serotypes, Infantis was the most prevalent serotype, followed by Senftenberg, Enteritidis, Kedougou, Tyhimurium, Heidelberg, Kentucky, Ohio, and Arizona. The majority of these serotypes were isolated from infants, while others were identified in stools from diarrheic adults, particularly of *Salmonella enterica* serotypes Enteritidis and Typhimurium, in the Skikda region (northeastern Algeria). Hawwas et al. [31], in their research in the Suez Canal area in Egypt, aimed to ascertain the occurrence of *Salmonella* serovars in sheep and goats and evaluate their potential zoonotic risk to humans. Out of 100 human stool samples tested, 13% exhibited positive results for *Salmonella*. The study also underscores the significance of apparently healthy and diarrheic sheep and goats as reservoirs and potential sources of human infection, with virulent *Salmonella* serovars in the Suez Canal area. According to Oueslati et al. [19], the study reported the predominance of three serotypes represented by Enteritidis, Typhimurium, and Kentucky in isolated *Salmonella* from hospitalized patients with gastroenteritis in Great Tunisia between 2010 and 2020.

The monitoring of the *Salmonella* serotypes that are circulating among humans in North Africa is important public health information about the regional epidemiology of salmonellosis. The knowledge of the genetic variety of *Salmonella* serotypes in different food sources is crucial for devising targeted intervention strategies. The present situation of human salmonellosis in North Africa shows the relationship between the prevalence of infections and food-related factors [7]. Therefore, it is crucial for public health authorities, regulatory agencies, and the food industry to partner to implement effective preventive measures, ensure food safety, and safeguard public health in the region. These measures consisting thorough research, quantitative microbial risk assessment, and multilateral measures to reduce the risks of salmonellosis.

## 4. Source and Modes of Transmission to Humans in North African Countries

*Salmonella* is one of the most frequently encountered zoonotic pathogens that are responsible for foodborne illnesses all over the world. *Salmonella* mostly attacks humans when they eat animal-based foods like beef, poultry, pork, eggs, and milk that are contaminated with the pathogen. In addition, contact with animals, for example, reptiles and turtles, can lead to non-typhoidal *Salmonella* transmission [32,33]. Fruits and vegetables tainted with animal feces or cross-contaminated with *Salmonella* from animal food during meal preparation have also been implicated in outbreaks of salmonellosis in humans [34,35]. Although whole-genome sequencing (WGS) is labor-intensive and expensive, it remains the most reliable method for genotyping *Salmonella* isolates and analyzing their epidemiological relationships. Other techniques, including Pulsed-Field Gel Electrophoresis (PFGE), Multi-Locus Sequence Typing (MLST), and Repetitive-Element PCR (rep-PCR), can also provide useful data, but they yield less comprehensive information compared to WGS.

The proper information concerning the *Salmonella* occurrence in food as well as risk factors associated with human behavior (food handling, food storage, hygiene, meal preparation practices) and human health and immunity are important for the understanding of the possible foodborne infections in North African countries [36]. A meta-analysis by Al-Rifai et al. [9], which was based on the testing of 252,831 individuals, revealed that 6356 of them were positive for non-typhoidal *Salmonella*. The collected *Salmonella* prevalence in the Middle East and North Africa was calculated at 6.6% (95% confidence interval (CI): 5.4–7.9%), the highest pooled *Salmonella* prevalence measures were in Morocco (1997–2012), followed by Tunisia (1988–2009). Their 2019 study systematically reviewed the prevalence of non-typhoidal *Salmonella* in foods in the Middle East and North Africa, and it found that the overall prevalence is 8.8%. In animal-based foods, the prevalence was 9.0%, while the fishery products and plant-based foods both had the pooled prevalent rates of 22.9% and 0.4%, respectively. The most common serotypes in the area were Typhimurium (28%), Enteritidis (23.6%), and Kentucky (20.3%). Nevertheless, there is a gap in understanding the occurrence of non-typhoidal *Salmonella* in the North African countries’ food chain, and limited efforts have been made for molecular analyses to determine the sources of this major pathogen [7].

*Salmonella* can be transmitted to humans through direct as well as indirect contact. *Salmonella* can spread indirectly through different sources such as food, water, and the surrounding environment. It has the potential to infect lots of foods such as poultry products, eggs, meat, dairy products, fruits, and vegetables. Consumption of such foods may lead to infection. Close physical contact with animals or animal products in the course of work is the main reason for direct transmission which is more probable in people with such occupations [37,38]. Direct transmission is more likely in people engaging in careers that involve close contact with animals or animal-derived products. Examples of such professions include farmers, veterinarians, workers in the abattoirs, poultry operators, and butchers. Another source of infection is through direct contact with infected pets, especially puppies or kittens displaying diarrhea from *Salmonella* [39]. There are multiple routes through which zoonotic diseases can spread across humans and animals, but the most typical routes include the consumption of infected meat, eggs, or milk [33].

**Figure 2:**
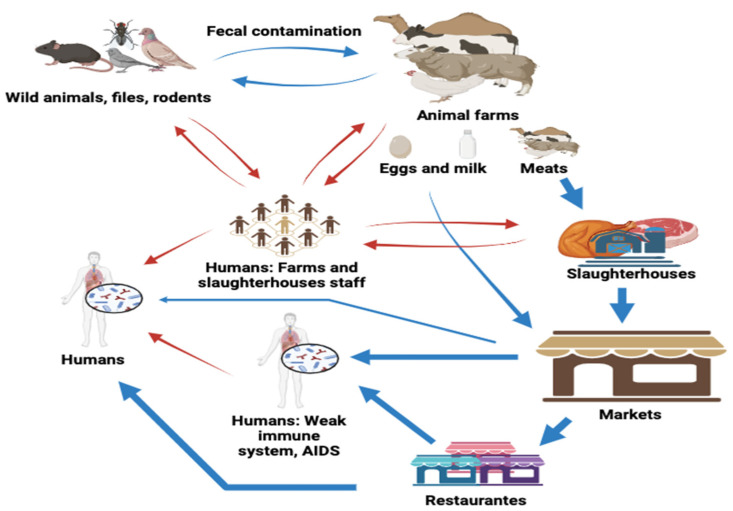
The figure displays both the direct and indirect routes of *Salmonella* transmission to humans in North Africa. Red arrows: direct routes; Blue arrows: indirect routes. Created using Biorender.com (accessed on 12 June 2024).

In North Africa, different social factors that play a role in malnutrition include poverty, poor access to clean water, and inadequate food supplies. Malnutrition weakens the immune system therefore exposing the body to diseases such as salmonellosis. Furthermore, illnesses such as AIDS increase susceptibility to *Salmonella* infections. The immune system has decreased efficiency in combatting pathogens in HIV/AIDS patients, making such patients potential carriers of *Salmonella* and sources of transmission [40]. These unique conditions in the countries of North Africa prove the necessity of understanding the relationship between consumer health and immunity concerning salmonellosis. *Salmonella* prevention and control measures should target these risk factors to implement targeted prevention and control measures effectively. As shown in Figure 2, there are possible pathways of *Salmonella* transmission to humans in North Africa.

Table 1 summarizes a recent research study on *Salmonella* frequency in North African food. Research studies performed throughout North African countries disclose *Salmonella* contamination in various food products, especially those of poultry origin [7]. In Algeria, the study highlights *Salmonella*’s existence in poultry products like eggs as well as chicken meat, sourced from mini-markets, wholesalers, and street vendors [25,41]. In addition, an association between *Salmonella* serotypes impacting humans and poultry suggests that poultry manufacturing is a factor in *Salmonella* dissemination [23].

Similarly, Egypt has detected *Salmonella* in poultry products like chicken hamburgers, filets, whole carcasses, raw egg yolks as well as eggshells, together with contamination in poultry meat throughout various areas [42,43]. Furthermore, *Salmonella* has been found in buffalo meat as well as dairy products in Egypt [27].

In Morocco, meat consumption, especially chicken, has caused foodborne diseases, with *Salmonella* found in poultry products, eggs, beef, and dairy [44,45]. Seafood, particularly along the North shore, can be contaminated with *Salmonella* [46].

Libya experiences *Salmonella* contamination in milk, dairy products, minced meat as well as beef hamburgers [13,28,47]. In Tunisia, *Salmonella* has been found in clams, cow milk, as well as various other food products, demanding detailed control actions [48]. These findings across North Africa highlight the immediate demand for robust surveillance and control measures to alleviate the risks posed by *Salmonella* contamination in food products. Thorough research along with enhanced control actions are essential to deal with the challenges related to *Salmonella* throughout different food resources, highlighting the considerable role of poultry manufacturing in *Salmonella* transmission.

The existence of *Salmonella* alone might not be enough to necessitate control procedures. Rather an extensive risk analysis that considers all relevant risk factors with their interactions is essential to justify applying control procedures successfully [7]. This strategy ensures that interventions are targeted and proportional to the degree of risk posed by *Salmonella* contamination. By considering aspects such as the source of contamination, transmission paths, and possible health impacts a thorough risk assessment provides the basis for educated decision-making regarding control steps [9]. Initiatives in Morocco underscore the value of rigorous surveillance, while in Libya the emphasis is on boosting food security in meat products. Continuous surveillance, especially for Enteritidis, is critical in Tunisia.

**Table 1:** The presence of salmonella in food across North African countries.

Country	Tested Food Samples (Total Number)	% (Out of Total Number) of NTS Positive Samples	*Salmonella* Serotypes (%) *	Confirmation Methods (If PCR Targets Genes) and References
Algeria	Chicken meats (*n* = 60)	50	ND	API 20E identification kit [26]
	Chicken meats (*n* = 70)	57.14	ND	MALDI-TOF MS [25]
	Frozen beef liver (*n* = 50)	4	ND	Biochemical test [49]
	Chicken Liver (*n* = 25)	4	Kentucky (100)	MALDI-TOF MS [23]
	Fruit and vegetable (*n* = 181)	0 (not detected)	ND	ND [33]
	Eggs (*n* = 45)	4.4	Bradford (100), Entritidis (0)	API-10S identification kit [50]
	Eggs (*n* = 180)	7.22	ND	Biochemical test [41]
	Beef (*n* = 190)	22.52	ND	PCR technique (dNTPs) [51]
	Mutton (*n* = 251)	12.74	ND	
	Dairy products (*n* = 310)	ND	ND	Biochemical test [52]
	Raw red meat and meat products (*n* = 144)	23.6	Agona (1.6), Albany (3.1), Altona (12.5), Anatum (14.0), Corvallis (7.8), Enteritidis (7.8), Hadar (1.6), Heidelberg (4.7), Indiana (4.7), Infantis (1.6), Kedougou (1.6), Lexington (1.6), Liverpool (1.6), Mbandaka (4.7), Montevideo (6.3).	API 20E identification kit [14]
Egypt	Chicken burger (*n* = 30)	10	Typhimurium (0), Infantis (100), Kentucky (0)	PCR technique (*hilA*, *invA*, *stn*) [43]
	Chicken filet (*n* = 30),	23.33	Typhimurium (28.57), Infantis (42.86), Kentucky (28.57)	
	Chicken luncheon (*n* = 30), Chicken nuggets (*n* = 30), and Chicken panne (*n* = 30)	0	ND	
	Market raw milk (*n* = 30)	6.7	Enteritidis (7.1), Typhimurium (7.1), Virchow (7.1), Larochelle (0), Apeyeme (0)	Biochemical test [4]
	Bulk tank milk (*n* = 30)	6.7	Enteritidis (7.1), Typhimurium (7.1), Virchow (0), Larochelle (7.1), Apeyeme (0)	
	Pasteurized milk (*n* = 30)	0	ND	
	Kariesh cheese (*n* = 30)	16.7	Enteritidis (14.9), Typhimurium (7.1), Virchow (14.9), Larochelle (7.1), Apeyeme (7.1)	
	White soft cheese (*n* = 30)	3.3	Enteritidis (0), Typhimurium (7.1), Virchow (0), Larochelle (0), Apeyeme (0)	
	Raw/fresh food (*n* = 300)	7.67	ND	
	Ready to eat/drink food (*n* = 56)	8.93	ND	MALDI-TOF MS [53]
	Buffalo meat (*n* = 100)	25	Enteritidis (20.8), Typhimurium (17), Montevideo (11.3), Rissen (9.4), Infantis (9.4), Virchow (7.5), Essen (5.7), Anatum (3.8), Dublin (3.8), Tsevie (3.8), Chester (1.9), Derby (1.9), Papuana (1.9), Saintpaul (1.9).	PCR technique (*invA*) [27]
	Fresh poultry (*n* = 60)	10	Enteritidis (3.3), Typhimurium (3.3), Kentucky (3.3), Paratyphi A (6.7)	PCR technique (*ompC*) [54]
	Frozen poultry (*n* = 30)	3.3		
	Fresh beef (*n* = 60)	11.7	Enteritidis (3.3), Typhimurium (6.7), Kentucky (0), Paratyphi A (3.3)	
	Frozen beef (*n* = 30)	3.3		
	Fresh vegetables and ready-to-eat salads (*n* = 121)	0 (not detected)	ND	ND [55]
	Raw chicken meat (*n* = 100)	5	Typhimurium (60.0), Enteritidis (40.0).	MALDI-TOF MS [56]
	Poultry products (*n* = 75)	6.6	Enteritidis (40), Typhimurium (40), Kentucky (20)	PCR technique (*invA*) [57]
	Fresh chicken meat (*n* = 200)	3.5	Enteritidis (14.3), Typhimurium (71.4), Kentucky (14.3).	PCR technique (*invA*) [58]
	Ready-to-eat chicken meat (*n* = 100)	ND		
	Raw egg yolk (*n* = 30)	ND	Enteritidis (3.1), Typhimurium (0), Kentucky (41.5), Other types (55.4)	API 20E identification kit [42]
	Eggshell (*n* = 30)	ND		
	Mixed chicken meat samples (*n* = 62)	60		
	Chicken skin (*n* = 22)	64		
	Chicken carcasses (*n* = 50)	16	Enteritidis (37.4), Typhimurium (30.1), Kentucky (10.8), Muenster (8.4), Virchow (4.8), Anatum (4.8), Haifa (1.2), Other types (2.4)	API 20E identification kit [59]
	Frozen beef (*n* = 160)	2.5	Enteritidis (32.1), Typhimurium (41.5), Infantis (20.8)	PCR technique (*ompC*) [60]
	Fresh beef (*n* = 80)	18.7		
	Beef carcasses (*n* = 240)	8.3		
	Chicken breast (*n* = 160)	1.25		
	Chicken legs (*n* = 160)	7.5		
	Raw milk (Buffalo) (*n* = 240)	3.3		
	Raw milk (Cow) (*n* = 240)	1.6		
	Cheese (Kareish) (*n* = 120)	2.5		
	Cheese (Domiati) (*n* = 120)	0.83		
	Yogurt (*n* = 80)	ND		
	Frozen chicken breast filets (*n* = 25)	52	Enteritidis (100)	PCR technique (*Rfbj*, *Flic*, *Fljb*, *Sdf*) [61]
	Frozen chicken legs (*n* = 25)	36	Enteritidis (100)	
	Minced frozen meats (*n* = 25)	20	Kentucky (100)	
Morocco	Egg content (*n* = 290)	2	ND	Biochemical test [62]
	Eggshell (*n* = 290)	0	ND	
	Broiler Chicken Meat (*n* = 540)	7.40	ND	API 20E identification kit [45]
	Chicken breast cut (*n* = 128)	12.5	Hadar (23.80), Typhimurium(19.05), Chester (19.05) Schwarzengrund (14.28), Kentucky (9.52), Bredeney (9.52), Saintpaul (4.76)	PCR technique (*invA*) [5]
	Viscera (liver, gizzard, heart) (*n* = 197)	7.92		
	Cut meats (skewers samples) spiced (*n* = 101)	5.94		
	Cut meats (skewers samples) not spiced (*n* = 80)	11.25		
	Minced meat (*n* = 55)	12.73		
	Chicken sausages (*n* = 54)	11.11		
	Traditional chicken mortadella (*n* = 5)	40		
	Shellfish (*n* = 150)	12.7	Chester (73.7), Hadar (15.8), Typhimurium (5.7), Kentucky (5.7)	PCR technique (*invA*) [63]
	Traditional cheese (*n* = 51)	5.9	Typhimurium (6.2), Enteritidis (4.2), Kentucky (22.9), Montevideo (6.2), Agona (16.7), Reading (12.5), Corvallis (8.3), Saintpaul (8.3), Israel (2.0), Hadar (2.0), Branderup (2.0)	API 20E identification kit [44]
	Minced meat (*n* = 138)	12.3		
	Sausage (*n* = 20)	5		
	Chicken (*n* = 86)	20		
	Turkey (*n* = 17)	52.9		
	Milk and other derivatives (*n* = 152)	ND.		
	Turkey sausages (*n* = 60)	23.3	Typhimurium (5.9), Agona (2.9), Saintpaul (2.9), Mbandaka (11.8), Montevideo (8.8), Livingstone (2.9), Corvallis (23.5), Kentucky (17.6), Bovismorbificans (5.9), Anatum (2.9), Give (11.8), Muenster (2.9)	PCR technique (*invA*, *spvC*) [64]
	Beef sausages (*n* = 60)	15		
	Artisanal sausages (*n* = 36)	30.6		
	Cereal products (*n* = 60)	ND	-	API 20E identification kit [65]
	Chicken meat, eggs, and visceral organs (*n* = 432)	0.7	Hadar (9.1), Corvallis (18.2), Mbandaka (18.2), Ouakam (18.2), Tm var. cop (9.1), Virchow (18.2), Altona (9.1).	API 20E identification kit [66]
	Mussels (*n* = 279)	10	Kentucky (57.1), Blockley (42.9), Senftenberg (0)	PCR technique (*invA*) [67]
	Cooked meat (*n* = 2952)	0.7	Anatum (3.8), Bareilley (1.0), Berta (1.9), Blokley (10.4), Brenderup (6.6), Bredeney (12.3), Enteritidis (2.8), Hadar (3.8), Infantis (23.8), Kiambu (5.7)	API 20E identification kit [46]
	Sausages (*n* = 2052)	0.1		
	Chicken meat (*n* = 1200)	0.4		
	Pastry (*n* = 2232)	0.2		
	Chopped meat (*n* = 196)	2.4		
	Sea food (*n* = 562)	1.8		
	Spices (*n* = 80)	1.3		
	Beef meat (*n* = 2122)	3.5	Labadi (1.9), MBandaka (7.6), Montevideo (3.8), Typhimurium (8.5), Salamae type II (1.0), Non-typeable (2.8)	
	Raw ground beef (*n* = 150)	2	Entritidis (33.3), Typhimurium (33.3).	API 20E identification kit [68]
	Fresh sausage (*n* = 100)	4	Anatum (33.3), Bareilly: (14.3)	
	Chicken breast, legs, liver, gizzard (*n* = 576)	10	Typhimurium (40.4), Newport (26.3), Montevideo (17.5), Heidelberg (15.8)	Biochemical test [69]
	Vegetable samples (*n* = 50)	2	Arizona (100)	API 20E identification kit [70]
Libya	Raw cow milk (*n* = 56)	16	ND	PCR technique (*16S rDNA*) [13]
	Fermented raw milk (*n* = 18)	11	ND	
	Milk powder (*n* = 18)	0	ND	
	Maasora cheese (*n* = 13)	22	ND	
	Ricotta cheese (*n* = 13)	46	ND	
	Ice cream (*n* = 13)	0	ND	
	Minced meat (*n* = 50)	6	Typhimurium (33.3), Enteritidis (Hamad and Saleh, 2019) (3)	PCR technique (*invA*) [47]
	Beef burger (*n* = 50)	4	Typhimurium (50), Enteritidis (50), Inganda (0)	
	Uncooked chicken burger (*n* = 56)	12.5	ND	API 20E identification kit [28]
	Cooked spiced chicken burger (*n* = 64)	1.6	ND	
Tunisia	Chicken products (*n* = 1288)	5.7	Enteritidis (22), Kentucky (19), Anatum (18), Infantis (16), Mbandaka (8), Zanzibar (8), Hadar (5), Agona (4)	API 20E identification kit [15]
	Clams (*n* = 20)	35	Irenea (28.6), London (28.6), Enteritidis (14.3), Poona (14.3), Brandcaster (14.3)	PCR technique (*siiA*) [48]
	Chicken (*n* = 97)	28.9	Enteritidis (64.3), Kentucky (35.7)	
	Cow’s milk (*n* = 80)	12.5	Kentucky (80), Anatum (20)	
	Cooked dishes (*n* = 150)	21.3	Zanzibar (0.08), Kentucky (28.0), Manchester (12.0), Schwarzengrund (0.08), Bredeney (4.0), Altona (12.0), Anatum (20.0), Amsterdam (4.0), Orion (4.0)	API 10S identification kit [29]
	Raw milk (*n* = 93)	33.3		
	Dairy products (*n* = 22)	22.7		
	Vegetables salad (*n* = 70)	12.8		
	Seafood (*n* = 46)	23.9		
	Raw poultry meat including (*n* = 45)	60		
	Cakes (*n* = 41)	26.8		
	Salami and sausage (*n* = 20)	25		
	Raw red meat (*n* = 13)	35.5		
	Chicken carcasses (*n* = 50)	16	Enteritidis (100)	API 20E identification kit [71]
	Cuts of beef (*n* = 144)	29.8	Typhimurium (35.0), Kentucky (17.5), Suberu (15.0), Newlands (8.8), Zanzibar (13.8), Orion (7.5), Enteritidis (3.8), Neumuenster (1.3)	PCR technique (*invA*) [72]
	Raw chicken (*n* = 60)	48.3		
	Portions of minced meat (*n* = 56)	10.7		
	Cuts of lamb (*n* = 33)	6		
	Merguez (sausages) (*n* = 10)	ND		
	Fish (*n* = 12)	ND		

* The percentage (%) of *Salmonella* serotypes is calculated from the positive samples (isolated target bacteria). ND: Not determined.

## 5. Virulence Factors

Virulence genes in bacteria, including *Salmonella* are essential in causing foodborne diseases. These genes help the microorganism to adhere to cells and tissue, invade the tissue, and also avoid detection and destruction by the host’s immune system. They play an essential function in *Salmonella*’s capacity to cause infections [73]. Studying these genes is crucial for establishing efficient strategies to regulate as well as avoid *Salmonella* relevant foodborne diseases, highlighting the importance of recognizing the molecular systems driving bacterial pathogenicity coupled with stress responses [74].

Studies utilizing animal models have demonstrated that *Salmonella* strains harboring specific virulence genes, including those associated with adhesion, invasion, and intracellular survival, are more prone to inducing severe infections and higher mortality rates compared to strains lacking these genes [75,76,77]. For instance, research has shown that *Salmonella* strains with intact virulence gene clusters, such as *Salmonella* pathogenicity islands (SPIs), display heightened virulence in animal models, resulting in more severe disease outcomes [78]. Furthermore, the epidemiological studies revealed correlations between the detection of some virulence genes in Salmonella isolates and cases of severe salmonellosis outbreaks in humans [79,80]. For instance, the association between specific virulence factors like the *Salmonella* enterotoxin gene (*stn*) is correlated to increased severity and hospitalization morbidity among infected patients [81]. In addition, whole-genome sequencing has also aided in the identification of the genetic determinants of *Salmonella* virulence basis of pathogenicity of *Salmonella* and its effects on the severity of the illness [82]. By analyzing the complete genetic composition of *Salmonella* isolates, researchers can identify virulence genes and evaluate their prevalence and distribution across different strains. These investigations have unveiled correlations between the presence of certain virulence genes and the ability of *Salmonella* strains to cause invasive infections or evade host immune responses [81]. Furthermore, in vitro and ex vivo experiments have elucidated the roles of specific virulence genes in *Salmonella* pathogenesis. Through manipulation of gene expression or deletion of virulence genes from *Salmonella* strains, researchers can assess their contributions to bacterial virulence and disease progression [83]. These experimental methods offer to confirm the value of virulence genes in assisting in *Salmonella* infection coupled with affecting disease severity.

The absence of virulence genes in *Salmonella* isolates from food explains why some strains rarely cause salmonellosis [79]. These genes encode factors aiding *Salmonella* invasion and survival within host cells, and their reduction in food strains diminishes pathogenicity [73]. Consequently, ingestion of such strains often results in milder illness or asymptomatic infection. Additionally, lacking key virulence genes may impede *Salmonella* ability to colonize and replicate in humans, further reducing disease likelihood [79]. Thus, the existence or lack of virulence genes in foodborne *Salmonella* is critical in establishing their possibility to influencing disease severity. Recognizing the virulence genes of foodborne *Salmonella* is important for analyzing hygienic threats and also applying targeted treatments to suppress foodborne ailments [19].

*Salmonella* spp.’s pathogenicity relies on virulence factors encoded by genes organized into “pathogenicity islands” (SPI) [84]. There are five well-defined SPIs with SPI1 as well as SPI2 inscribing type III secretion systems [85,86]. SPI1 facilitates host cell invasion and inflammation, while SPI2 is crucial for intracellular survival and replication within phagocytes, contributing to systemic spread [87]. The *invA* gene in SPI1 exists in many *Salmonella* strains and SPI2 also consists of the *spiC* gene necessary for encoding structural components and secretion, essential for *Salmonella*’s virulence [88]. Wang et al. [89] propose that *spiC* crucial for *Salmonella* infection does not influence flagella filament elements. While SPI3 exists in all lineages, the distributions of SPI4 and also SPI5 are unclear [78]. SPI4 plays a role in preliminary communications with the digestive tract epithelial cells and also long-term determination, containing the *orfL* gene for intramacrophage survival [90]. SPI5 participates in different infection processes with *pipD* [78]. *Salmonella*’s extra virulence elements outside SPI, like the *Salmonella* virulence plasmid (*spvRABCD*), boost systemic spread coupled with replication in extraintestinal sites [84,91]. The polyamine constant, or the concentration of polyamines in food, is particularly high in certain fermented, aged, and plant-based foods. These compounds play vital roles in both normal cellular functions and bacterial survival. For pathogens like *Salmonella*, the high polyamine levels in food can enhance survival in the host digestive system and potentially increase virulence, making it more difficult to combat infections. Understanding the relationship between polyamine content in food and bacterial pathogenesis is crucial for developing better strategies to control foodborne infections and improve public health outcomes [92].

Table 2 shows the distribution of virulence genes among serotypes in food and human samples across North African countries. The possession of specific virulence genes correlates with variations in illness rates among different serotypes of *Salmonella* [93]. These genes contribute to the pathogenicity of certain strains, influencing their ability to cause severe illness compared to others [94]. The study by Nouichi et al. [95] in Algerian slaughterhouses analyzed 84 *Salmonella* isolates from sheep and cattle, identifying 10 serotypes and assessing the presence of virulence genes (*invA*, *pefA*, *sefA*, *pipB*, *sseC*, *ssaP*, *spvC*, and *iroB*). Different gene distribution patterns were observed, with *iroB* being the most prevalent (77.4%), while *sefA* was absent. *PefA* and *spvC* were detected in Typhimurium, and the remaining four genes were present in 61.9% of isolates. The occurrence of virulence genes did not significantly vary based on serotypes, animal species, or sample type; thus, it can be said that the pathogenic *Salmonella* strains that are transmissible are present in Algerian slaughterhouses. Moreover, Helal et al. [96] characterized the *Salmonella* isolates from different poultry organs in Egypt, and they were able to identify the specific amplicons for the virulence-encoding genes in all tested *Salmonella*. The randomized study conducted by Diab et al. [97] examined the virulence genes in the non-typhoidal *Salmonella* isolates from both humans and animals in Egypt, and it was found that the occurrences of *invA* and other genes such as *stn*, *spvC*, and *hilA* were very high. Reference [56] characterized the *Salmonella* isolates that were obtained from chicken meat in Egypt and revealed the distinct gene profiles for Enteritidis and Typhimurium. In the study by Ed-Dra et al. [98] in Morocco, the pathogenicity of 34 *Salmonella* isolates obtained from sausages was assessed and the complete set of six virulence genes (*orgA*, *sitC*, *sipB*, *spiA*, *iroN*, and *sifA*) found in all the isolates, emphasizing the public health risk that accompanies *Salmonella* contamination of Moroccan sausages.

Gritli et al. [71] examined 24 Enteritidis isolates in Tunisia, identifying six virulence genes, predominantly associated with SPIs. While SPI-1, 2, and 3 genes were consistently present, SPI-4 and 5 genes showed variable occurrence rates. The Enteritidis-specific virulence gene *spvC* was amplified in 45.8% of isolates. Hassena et al. [48,99] conducted genomic screenings of *Salmonella* isolates in Tunisia, revealing exclusive virulence gene profiles in Enteritidis strains. Diverse virulence profiles were identified, even within the same serovar, with eight distinct virulence profiles or virulotypes observed. Oueslati et al. [15,24] conducted genomic assessments of *Salmonella* isolates, identifying predominant virulotypes in different settings, associated with invasion, biofilm formation, intracellular survival, and enteropathogenic functions. A subsequent investigation of 61 strains from gastroenteritis patients in Greater Tunisia identified *invA*, *mgtC*, *sirA*, *gipA*, and *pagK* (32.8%) as the most prevalent. All strains showed positive attributes for *invA*, *pagK*, *mgtC*, and *sirA* genes associated with invasion, biofilm formation, intracellular survival, and enteropathogenic functions, while testing negative for *spvC*, *trhH*, *SEN1417*, *sipA*, *sipD*, and *sopD* virulence genes [29].

These virulence genes were found in the *Salmonella* isolates from different food sources, as well as from the hospitalized patients suffering from gastroenteritis in Tunisia and other North African countries (Table 2), indicating their pathogenicity during an outbreak. This underlines the need for comprehensive studies on all processing stages of food products of animal and plant origin as well as supermarkets and restaurants where a lot of food preparation occurs in these countries.

## 6. Antibiotic Resistance

The general overuse of antibiotics by humans and animals has greatly worsened antibiotic resistance, which in turn has caused the worldwide increase in multidrug resistant (MDR) *Salmonella* in different food products [2,37]. The emergence of MDR *Salmonella* has been closely linked to the misuse of antibiotics. These resistant strains are increasingly found in food sources, complicating efforts to ensure food safety and combat foodborne illnesses. This surge in drug resistance, notably affecting critical antibiotics like β-lactams, poses a challenge to combating bacterial infections in humans [82,100]. The invention of the extended-spectrum oxyimino cephalosporins in the 1980s was the first step towards the identification of the extended-spectrum β-lactamases (ESBLs), with the prominent types being *TEM*, *SHV*, and *CTX-M*. The increasing frequency of *CTX-M*-producing bacteria in the last decade has made the situation even more complicated, as these enzymes give both resistance and resistance to other drug classes at the same time [101,102,103,104]. This escalating resistance has profound implications for public health, food safety, and clinical medicine. The reduced efficacy of antibiotics necessitates the urgent development of novel antimicrobial agents and stricter regulation of antibiotic use in both human and veterinary practices. Additionally, continuous surveillance of resistance patterns and molecular characterization of resistant strains are critical for tracking the spread of MDR *Salmonella* and other pathogens.

In Algeria, the ESBLs in non-typhoidal *Salmonella* among humans were first detected in 1994 (Rahal and Reghal, 1994), yet the data on the prevalence in animals and humans were not available until 2017. Djeffal et al. [23] have been researching this and filled the gap by exploring the contamination levels, the antimicrobial resistance, the possibility of ESBL production, and the genetic relationship of the strains in the chicken farms, the slaughterhouses, and those that cause human illness in northeastern Algeria. They discovered a close genetic relationship between human and poultry strains, which implies that poultry farming played a big role in the spread of multidrug-resistant *Salmonella*.

The researchers Merati and Boudra, ref. [41], isolated 13 *Salmonella* isolates in the eggs of Tiaret Province, Algeria, which have found high resistance to common antibiotics. The study showed that all the isolates tested were completely resistant to antibiotics like amoxicillin, clavulanic acid, ampicillin, nalidixic acid, and erythromycin. Street vendors obtained the highest *Salmonella* detection rate, highlighting antibiotic resistance.

In Algeria, Mezali and Hamdi, ref. [14], reported the emergence of ‘ACSSuT’-resistant Typhimurium strains in raw meat in 2012, marking a concerning development in antimicrobial resistance. Among 62 *Salmonella* isolates, 56 (90%) exhibited resistance to at least one antimicrobial agent, with 32% demonstrating multidrug resistance, particularly against sulphonamides (87%). Resistance rates were comparatively lower for nalidixic acid, streptomycin, and tetracycline, while pefloxacin resistance was noted at 4.84%. The study identified fourteen different resistance patterns, including the ‘ACSSuT’ penta resistance pattern observed in three Typhimurium strains. Furthermore, Nouichi et al. [51] reported the detection of ACSSuT-resistant Typhimurium strains in bovine and ovine samples in Algiers in 2018, underscoring the persistence and spread of antimicrobial resistance in the region.

In a study by Moawad et al. [54] in Egypt, antibiotic resistance profiles of 15 *S. enterica* isolates from raw chicken and beef in northern Egypt revealed ESBL and *AmpC* β-lactamase genes, including *bla_CTX-M_* (73.3%), *bla_TEM_* (73.3%), and *bla_CMY_* (13.3%). Plasmid-mediated quinolone resistance genes were also identified, with *qnrA* in 33.3%, *qnrB* in 20.0%, and *qnrS* in 6.7% of isolates. Abd-Elghany et al. (2015) reported resistance rates among 166 *Salmonella* isolates from chicken meat, with 92.8% exhibiting multidrug resistance.

In Libya, according to the study by Garbaj et al. [13], all nine *S. enterica* isolates tested were resistant to amoxicillin, bacitracin, penicillin G, lincomycin, vancomycin, clindamycin, and cloxacillin. On the other hand, these same strains exhibited susceptibility to levofloxacin, doxycycline, and ciprofloxacin. Also, all the tested isolates scored 100% resistance to more than one antibiotic used in the study.

In Morocco, the highest percentage of resistance was found in 48 *Salmonella* isolates obtained from food products to the following antimicrobial agents: nalidixic acid (27.1%), sulfonamides (25%), amoxicillin (12.5%), amoxicillin/clavulanic acid (12.5%), trimethoprim (10.4%), cephalothin (4.2%), and chloramphenicol (2.1%) [44]. In another study by Bouchrif et al. [44], it was found that twenty nine percent of the isolates (30 out of 105) obtained from food products displayed resistance to at least one antimicrobial agent. Tetracycline resistance was the most prevalent (21%), followed by resistance to ampicillin (13%), amoxicillin-clavulanic acid (9%), streptomycin (7%), chloramphenicol (4%), and nalidixic acid (3.8%). Notably, none of the isolates exhibited resistance to third-generation cephalosporins or fluoroquinolones like ciprofloxacin. Multidrug resistance (MDR) was identified in 9.5% of the isolates, primarily observed in Typhimurium DT104 with the R-type ACSSuT and Hadar. Multiple research investigations carried out in Morocco revealed resistant strains found in various food sources like table eggs [62], sausages [64], broiler chicken meat [45], and shellfish [63].

Hassena et al. [46] were the first report that detected the occurrence of the *AmpC* FOX and EBC gene families, as well as the *qnrD* gene in a foodborne pathogen in Tunisia. Their study used the PCR technique to check 38 isolates of *Salmonella* for genes responsible for resistance to cefotaxime. These genes were *bla_TEM_*, extended-spectrum beta-lactamases (*bla_CTX_* and *bla_OXA_*), *AmpC* beta-lactamases, FOX, MOX, DHA, ACC, CIT, and EBC. In this regard, one of the Kentucky isolates isolated from milk samples harbored the *AmpC* gene (FOX), while the same serotype isolated from chicken samples contained the EBC *AmpC* determinant. All the isolates that presented nonsusceptibility to at least one of the analyzed antimicrobials harbored the *bla_TEM_* gene. Additionally, the researchers investigated isolates with reduced susceptibility to fluoroquinolones for plasmid-mediated quinolone resistance determinants. They identified three qnr genres which included *qnrB*, *qnrD*, and *qnrS* in four isolates, two were isolated from milk samples, and the other two were isolated from chicken samples. It is interesting to mention that *S.* Kentucky rarely causes salmonellosis in humans.

The study by Oueslati et al. [24] found a significant level of resistance in 64 *Salmonella* isolates obtained from broiler chickens. High resistance rates were recorded for nalidixic acid (82.85%), amoxicillin (81.25%), streptomycin (75%), and ciprofloxacin (75%). Notably, resistance to ertapenem (12.5%) was also identified. Out of the total isolated strains, 87.5% (56 out of 64) were categorized as multidrug-resistant (MDR). Among the MDR strains, three exhibited the production of ESBL, and another three showed cephalosporinase production. The *bla_CTX-M_* gene was detected in all three ESBL strains. Interestingly, the *qnrB* gene was not found in strains resistant to fluoroquinolones. In tetracycline-resistant strains, the *tetA* gene was amplified in 5% (2 out of 40) and the *tetB* gene in 2.5% (1 out of 40). Additionally, among the 20 trimethoprim-resistant strains, the *dfrA1* gene was amplified in five instances. However, none of the phenotypically colistin resistant strains showed amplification of the *mcr*-1, *mcr*-2, *mcr*-3, *mcr*-4, or *mcr*-5 genes.

Through studies assessing *Salmonella* isolates in human and food portion samples collected from Tunisia, researchers discovered that the constant evolution of multidrug-resistant strains may make management of the infections difficult in the future. In Algeria and Egypt, more focus has been placed on food products as carriers of antimicrobial-resistant *Salmonella*, giving support to the need for higher surveillance and a holistic approach. Altogether these observations underscore the importance of conscious prescription of antibiotics and adequate strategies to mitigate AMR for protecting public health in North African countries.

These findings point out the worries about the uncontrolled use of antibiotics in veterinary medicine, which highlights the necessity for stricter rules and the stricter enforcement of the laws governing antibiotic use in food animal production. The measures for controlling antibiotic use are necessary to stop the emergence and the spread of multidrug-resistant *Salmonella* strains, thus, protecting public health and the foodborne illnesses that are associated with antimicrobial resistance. Additionally, the natural antibiotic resistance in *Salmonella* aids in predictive models for behavior in chickens with native microflora, enhances accuracy in simulating growth, assessing food safety risks, and evaluating control measures [10,105,106]. This contributes to improved food safety practices and public health outcomes by reducing *Salmonella* contamination in poultry products.

## 7. Discussion

*Salmonella*, a Gram-negative bacterium of the Enterobacteriaceae family, has a slender shape. The main way of transmission to humans is by eating contaminated animal-derived foods like beef, poultry, pork, eggs, and milk [9,107]. In North African countries, surveillance for *Salmonella* is carried out at different levels. National public health agencies, like the Ministries of Health, control surveillance by obtaining and giving data on the cases that are reported by healthcare facilities nationwide [108]. Regional and local health authorities also take part in the process by keeping an eye on cases within their territories. The centralized and regional laboratories use microbiological techniques to confirm *Salmonella* infections, while the epidemiologists investigate the confirmed cases and find the sources of infection and the commonalities among the cases [95]. The stakeholder collaboration improves the data exchange and the coordination of the responses. Public awareness campaigns raise the issue of food hygiene and early symptom recognition. Capacity-building programs are the way to strengthen the surveillance networks, healthcare systems, and professional capabilities, which, in turn, will decrease the *Salmonella* transmission risk and will improve health outcomes effectively [109,110].

In Algeria, several researchers suggest that poultry products are the primary vehicles of *Salmonella*. A high prevalence is reported by some investigations, while others report no contamination, which propounds a geographical discrepancy. For instance, ref. [23] asserts that investigations reveal a close correlation between the human-involved *Salmonella* strains and poultry domesticated strains, pointing towards poultry production as a driver in the spread of *Salmonella*. Chicken-related products surface as the leading cause of foodborne salmonellosis in Egypt. Many research articles point to different serotypes of *Salmonella* that are present in chicken meat, beef, and dairy products, underscoring the potential risk these food items pose to consumers. Moroccan research shows that the poultry industry is associated with foodborne illnesses, especially *Salmonella*. Libya struggles to determine the causes of the outbreak, making it challenging to connect the cases to specific pathogens. Issues are raised about the presence of *Salmonella* in dairy and meat. Tunisia considers Enteritidis as the main cause of infections, therefore authorities in Tunisia are focusing on the surveillance and control of *Salmonella*, especially Enteritidis, in different foods.

The variation in the virulence genes among the different *Salmonella* serotypes in North African countries, as shown by the studies in Algeria and Tunisia, proves the difference in the amount of the key virulence factors. To illustrate, the *iroB* gene was the most common, and the *sefA* gene was not found in any of the tested strains in Algerian slaughterhouses. In addition, the distribution of virulence genes did not differ significantly based on the serovar, animal species, or sample type, which means the existence of transmissible pathogenic *Salmonella* strains in this case [95]. In Egypt, the molecular characterization studies showed that there were different virulence-encoding genes in the *Salmonella* isolates from poultry food. The naming of specific genes such as *pagC*, *msgA*, *spiA*, *invA*, *prgH*, *orgA*, *sipB*, *tolC*, *iroN*, *lpfC*, *pefA*, *sitC*, *sifA*, and *sopB* shows the complexity of the virulence profile of *Salmonella* strains in this region [96]. In Morocco, the examination of virulence genes in *Salmonella* isolates from sausages showed the presence of a complete set of six virulence genes (*orgA*, *sipB*, *sitC*, *spiA*, *iroN*, and *sifA*); thus, the possibility of contamination and its significance for public health [98].

Antibiotic-resistant *Salmonella* is a worldwide problem because of the misuse of antibiotics in humans and animals. The multidrug-resistant strains, especially the β-lactams resistance, create problems for the treatment. The Algerian studies demonstrate that the ESBL prevalence in *Salmonella* from humans and poultry is evidence of the inter-population strain links [104,105]. The resistance patterns seen in commercial eggs and raw meat samples are further proof of the fact that antibiotic resistance is everywhere in the country [14,39]. In Egypt, the investigation of antibiotic resistance profiles in *Salmonella* isolates from raw chicken and beef meat showed the presence of genes encoding ESBL and *AmpC* β-lactamase. The high rate of resistance to multiple antibiotics, including the third-generation cephalosporins and fluoroquinolones, emphasizes the need to deal with antibiotic resistance in foodborne pathogens [54]. Research in Libya, Morocco, and Tunisia also showed alarming levels of antibiotic resistance in *Salmonella* isolates obtained from different food sources. The fact that there is resistance to the commonly used antibiotics such as amoxicillin, nalidixic acid, and sulfonamides, which is a matter of concern for public health and the limited treatment options available [13,48,62,111]. The discovery of resistance genes like *AmpC* FOX, EBC, and *qnr* in Tunisian *Salmonella* strains shows the complexity of the resistance mechanisms in the region [48]. In addition, the detection of multidrug-resistant strains which are less susceptible to vital antibiotics like fluoroquinolones and third-generation cephalosporins makes it difficult to treat and control them.

Antibiotic resistance is a critical issue in the treatment of *Salmonella* infections, driven largely by the overuse and misuse of antibiotics. The emergence of resistant *Salmonella* strains has underscored the urgent need to identify new drug targets and develop novel therapeutic strategies. By focusing on *Salmonella*’*s* virulence factors, intracellular survival mechanisms, and metabolic pathways, researchers can work towards developing effective treatments to combat resistant strains. Furthermore, innovative approaches, including phage therapy, synthetic biology, and natural product discovery, could provide much-needed solutions to the growing problem of antibiotic resistance.

The comparison of the risks of *Salmonella* in North Africa with other zoonotic diseases such as COVID-19 shows significant differences in the epidemiological profiles and public health impacts [33]. Although *Salmonella* infections are widespread and can cause illness and death, especially among weak immune system groups, they generally lead to lower hospitalization and mortality rates than COVID-19 [78,112]. The worldwide pandemic caused by SARS-CoV-2 has resulted in global transmission, healthcare systems being overwhelmed, and a large number of morbidity and mortality worldwide. The COVID-19 hospitalization and death rates are much higher than those associated with *Salmonella* [50,113]. Although salmonellosis has a lower severity than COVID-19, both diseases need to be monitored, prevented, and controlled to reduce the risk to public health in North Africa and beyond.

In summary, this thorough discussion highlights the importance of ongoing research and monitoring in the investigation of the various routes of *Salmonella* transmission to humans in North African countries. Studies at the molecular level across the region have shown the diversity of *Salmonella* strains and their virulence profiles, thus, the importance of such studies for effective control and prevention strategies is stressed. These results show the difficulty of controlling *Salmonella* infections in the region. In addition, the discussion on antibiotic resistance makes it clear that there is a need for improved surveillance, responsible antibiotic use, and the development of comprehensive strategies to fight the potential threat of antimicrobial resistance in *Salmonella* strains. Proactive actions are the key to protecting public health and keeping effective treatment options for bacterial infections. Handling these problems as a team contributes to the total efforts to make North African countries a healthier and safer environment, where surveillance networks are of vital importance in controlling infections through a multi-sectoral approach that includes laboratory testing, epidemiological investigation, collaboration, public awareness, and capacity building; thus, protecting public health and reducing the burden of disease in the region.

## Figures and Tables

**Table 2:** Distribution of virulence genes among serotypes in food and human samples across North African countries.

Country	Geographical Distribution	Tested Food Samples or Human	Trend Over Time	*Salmonella* Serotypes (Total Number)	Virulence Genes (%) *	Techniques Used for Virulence Genes Detection and References
Algeria	El-Harrach and Hussein-Dey slaughterhouses in Algiers,	Sheep and cattle	Between 2013 and 2014	Muenster (*n* = 33)	*invA* (63.6), *pefA* (0), *sefA* (0), *pipB* (0), *sseC* (63.6), *ssaP* (63.6), *spvC* (63.6), *iroB* (63.6)	PCR technique [95]
				Kentucky (*n* = 13)	*invA* (76.9), *pefA* (0), *sefA* (0), *pipB* (0), *sseC* (38.7), *ssaP* (38.7), *spvC* (38.7), *iroB* (38.7)	
				Infantis (*n* = 12)	*invA* (91.7), *pefA* (0), *sefA* (0), *pipB* (0), *sseC* (66.7), *ssaP* (66.7), *spvC* (66.7), *iroB* (66.7)	
				Anatum (*n* = 11)	*invA* (81.8), *pefA* (0), *sefA* (0), *pipB* (0), *sseC* (72.7), *ssaP* (72.7), *spvC* (72.7), *iroB* (72.7)	
				Richmond (*n* = 4)	*invA* (100), *pefA* (0), *sefA* (0), *pipB* (0), *sseC* (100), *ssaP* (100), *spvC* (100), *iroB* (100)	
				Havana (*n* = 3)	*invA* (66.7), *pefA* (0), *sefA* (0), *pipB* (0), *sseC* (0), *ssaP* (0), *spvC* (0), *iroB* (0)	
				Typhimurium (*n* = 3)	*invA* (100), *pefA* (0), *sefA* (100), *pipB* (100), *sseC* (100), *ssaP* (100), *spvC* (100), *iroB* (100)	
				Montevideo (*n* = 3)	*invA* (100), *pefA* (0), *sefA* (0), *pipB* (0), *sseC* (66.7), *ssaP* (66.7), *spvC* (66.7), *iroB* (66.7)	
				Virginia (*n* = 1)	*invA* (100), *pefA* (0), *sefA* (0), *pipB* (0), *sseC* (0), *ssaP* (0), *spvC* (0), *iroB* (0)	
				Braenderup (*n* = 1)	*invA* (100), *pefA* (0), *sefA* (0), *pipB* (0), *sseC* (100), *ssaP* (100), *spvC* (100), *iroB* (100)	
Egypt	Different poultry farms located in El-Minufyia and El-Gharbia governorates	Liver, yolk sac and spleen	Between February 2017 to December 2017	Sinchem (*n* = 1), Gallinarum (*n* = 1), *S. enterica* subsp. Salamae (*n* = 1), Kentucky (*n* = 1), Entertidis (*n* = 1), Typhimurium (*n* = 1), Heidelberg (*n* = 1), Hydra (*n* = 1), Virchow (*n* = 1), Farsta (*n* = 1)	*pagC* (100), *msgA* (100), *spiA* (100), *invA* (100), *prgH* (100), *orgA* (100), *sipB* (100), *tolC* (100), *iroN* (100), *lpfC* (100), *pefA* (100), *sitC* (100), *sifA* (100), *sopB* (100), *spvB* (70), *cdtB* (30)	PCR technique [96]
	Different localities in the New Valley and Assiut Governorates	Raw milk, kareish cheese, Damietta cheese, yogurt, ice cream, animal fecal swabs, human fecal swabs and hand swabs	Between April 2020 and May 2021	Enteritidis (*n* = 4), Typhimurium (*n* = 4), Infantis (*n* = 4), Tsevie (*n* = 4), Larochelle (*n* = 4), Virchow (*n* = 3), Haifa (*n* = 3), Molade (*n* = 3), Heidelberg (*n* = 2), Essen (*n* = 2), Shubra (*n* = 1), Alfort (*n* = 1), Apeyeme (*n* = 1)	*invA* (100), *hilA* (94.4), *stn* (72.2), *spvC* (30.6)	PCR technique [97]
	Suez Canal Area	Humans, sheep, and goats	2021	Typhimurium (*n* = 13)	*invA* (61.5), *sopB* (92.3), *stn* (92.3), *spvC* (92.3)	PCR technique [31]
				Enteritidis (*n* = 12)	*invA* (25), *sopB* (100), *stn* (100), *spvC* (100)	
				Heidelberg (*n* = 4)	*invA* (0), *sopB* (100), *stn* (100), *spvC* (100)	
				Dublin (*n* = 5)	*invA* (40), *sopB* (100), *stn* (100), *spvC* (100)	
				Montevideo (*n* = 5)	*invA* (40), *sopB* (100), *stn* (100), *spvC* (100)	
				Saintpaul (*n* = 6)	*invA* (50), *sopB* (100), *stn* (100), *spvC* (100)	
				Anatum (*n* = 3)	*invA* (33.3), *sopB* (100), *stn* (100), *spvC* (100)	
				Tsevie (*n* = 1)	*invA* (100), *sopB* (100), *stn* (100), *spvC* (100)	
				Chester (*n* = 1)	*invA* (0), *sopB* (100), *stn* (100), *spvC* (100)	
				Essen (*n* = 2)	*invA* (0), *sopB* (100), *stn* (100), *spvC* (100)	
				Apeyeme (*n* = 1)	*invA* (100), *sopB* (100), *stn* (100), *spvC* (100)	
				Infantis (*n* = 1)	*invA* (100), *sopB* (100), *stn* (100), *spvC* (100)	
Morocco	Meknes city	Sausages	2018	Typhimurium (*n* = 2), Kentucky (*n* = 6), Saintpaul (*n* = 1), Corvallis (*n* = 8), Montevideo (*n* = 3), Mbandaka (*n* = 4), Bovismorbificans (*n* = 2), Give (*n* = 4), Anatum (*n* = 1), Livingstone (*n* = 1), Muenster (*n* = 1), Agon (*n* = 1)	*orgA* (100), *sipB* (100), *sitC* (100), *spiA* (100) *iroN* (100) *sifA* (100)	PCR technique [98]
Tunisia	Bab Saadoun, Tunis.	Chicken consumed in military cantines	February to June 2013	Enteritidis (*n* = 4)	*invE*/*A* (100), *ttrC* (100), *mgtC* (100), *spvC* (45.8) *sopB* (12.5), *siiD* (0)	PCR technique [71]
	Sfax city	Foodborne *Salmonella* isolates	ND	Enteritidis (*n* = 27)	*siiA* (100), *sopB* (96.3), *sopE* (100), *sopE2* (100), *cat2* (100), *safC* (100), *sefB* (100), *spvC* (92.6) *spvB* (92.6)	PCR technique [99]
				London (*n* = 8)	*siiA* (100), *sopB* (100), *sopE* (0), *sopE2* (100), *cat2* (100), *safC* (100), *sefB* (0), *spvC* (0) *spvB* (0)	
				Kentucky (*n* = 6)	*siiA* (100), *sopB* (100), *sopE* (0), *sopE2* (0), *cat2* (16.7), *safC* (100), *sefB* (0), *spvC* (0) *spvB* (0)	
				Havana (*n* = 3)	*siiA* (100), *sopB* (100), *sopE* (0), *sopE2* (0), *cat2* (0), *safC* (100), *sefB* (0), *spvC* (0) *spvB* (0)	
				Zanzibar (*n* = 2)	*siiA* (100), *sopB* (100), *sopE* (0), *sopE2* (100), *cat2* (100), *safC* (100), *sefB* (0), *spvC* (0) *spvB* (0)	
				Anatum (*n* = 2)	*siiA* (100), *sopB* (100), *sopE* (0), *sopE2* (100), *cat2* (100), *safC* (100), *sefB* (0), *spvC* (0) *spvB* (0)	
				Infantis (*n* = 1)	*siiA* (100), *sopB* (100), *sopE* (0), *sopE2* (100), *cat2* (100), *safC* (100), *sefB* (0), *spvC* (0) *spvB* (0)	
				Manchester (*n* = 1)	*siiA* (100), *sopB* (0), *sopE* (0), *sopE2* (100), *cat2* (100), *safC* (100), *sefB* (0), *spvC* (0) *spvB* (0)	
				Livingstone (*n* = 1)	*siiA* (100), *sopB* (100), *sopE* (0), *sopE2* (100), *cat2* (0), *safC* (100), *sefB* (0), *spvC* (0) *spvB* (0)	
				Gabon (*n* = 1)	*siiA* (100), *sopB* (100), *sopE* (0), *sopE2* (100), *cat2* (100), *safC* (0), *sefB* (0), *spvC* (0) *spvB* (0)	
	Northeast Tunisia	Broiler flocks	September 2019 and August 2020	Kentucky (*n* = 13)	*invA* (100), *spvC* (0), *hli* (53.8) *gipA* (61.5), *mgtC* (100), *trhH* (0), *sirA* (100), *pagK* (100), *sipA* (0), *sipD* (0), *sopD* (0), *SEN* (0)	PCR technique [24]
				Mbandaka (*n* = 12)	*invA* (100), *spvC* (0), *hli* (33.3) *gipA* (83.3), *mgtC* (100), *trhH* (0), *sirA* (100), *pagK* (100), *sipA* (0), *sipD* (0), *sopD* (0), *SEN* (0)	
				Anatum (*n* = 11)	*invA* (100), *spvC* (0), *hli* (36.4) *gipA* (72.7), *mgtC* (100), *trhH* (0), *sirA* (100), *pagK* (100), *sipA* (0), *sipD* (0), *sopD* (0), *SEN* (0)	
				Zanzibar (*n* = 10)	*invA* (100), *spvC* (0), *hli* (80) *gipA* (100), *mgtC* (100), *trhH* (0), *sirA* (100), *pagK* (100), *sipA* (0), *sipD* (0), *sopD* (0), *SEN* (0)	
				Enteritidis (*n* = 8)	*invA* (100), *spvC* (0), *hli* (75) *gipA* (87.5), *mgtC* (100), *trhH* (0), *sirA* (100), *pagK* (100), *sipA* (0), *sipD* (0), *sopD* (0), *SEN* (0)	
				Infantis (*n* = 2)	*invA* (100), *spvC* (0), *hli* (0) *gipA* (100), *mgtC* (100), *trhH* (0), *sirA* (100), *pagK* (100), *sipA* (0), *sipD* (0), *sopD* (0), *SEN* (0)	
				Indiana (*n* = 2)	*invA* (100), *spvC* (0), *hli* (50) *gipA* (100), *mgtC* (100), *trhH* (0), *sirA* (100), *pagK* (100), *sipA* (0), *sipD* (0), *sopD* (0), *SEN* (0)	
				Corvallis (*n* = 1)	*invA* (100), *spvC* (0), *hli* (0) *gipA* (100), *mgtC* (100), *trhH* (0), *sirA* (100), *pagK* (100), *sipA* (0), *sipD* (0), *sopD* (0), *SEN* (0)	
				Agona (*n* = 1)	*invA* (100), *spvC* (0), *hli* (0) *gipA* (100), *mgtC* (100), *trhH* (0), *sirA* (100), *pagK* (100), *sipA* (0), *sipD* (0), *sopD* (0), *SEN* (0)	
				Hadar (*n* = 1)	*invA* (100), *spvC* (0), *hli* (100) *gipA* (100), *mgtC* (100), *trhH* (0), *sirA* (100), *pagK* (100), *sipA* (0), *sipD* (0), *sopD* (0), *SEN* (0)	
				Montevideo (*n* = 1)	*invA* (100), *spvC* (0), *hli* (0) *gipA* (0), *mgtC* (100), *trhH* (0), *sirA* (100), *pagK* (100), *sipA* (0), *sipD* (0), *sopD* (0), *SEN* (0)	
				Cerro (*n* = 1)	*invA* (100), *spvC* (0), *hli* (100) *gipA* (100), *mgtC* (100), *trhH* (0), *sirA* (100), *pagK* (100), *sipA* (0), *sipD* (0), *sopD* (0), *SEN* (0)	
				Virginia (*n* = 1)	*invA* (100), *spvC* (0), *hli* (0) *gipA* (100), *mgtC* (100), *trhH* (0), *sirA* (100), *pagK* (100), *sipA* (0), *sipD* (0), *sopD* (0), *SEN* (0)	
	Great Tunisia	Hospitalized patients with gastroenteritis	Between 2010 and 2020	Enteritidis (*n* = 17)	*invA* (100), *mgtC* (100), *sirA* (100), *gipA* (76.5), *pagK* (100), *hli* (11.8), *trhH* (0), *spvC* (0), *sipA* (0), *sipD* (0), *sopD* (0), *SEN 1417* (0)	PCR technique [19]
				Typhimurium (*n* = 16)	*invA* (100), *mgtC* (100), *sirA* (100), *gipA* (62.5), *pagK* (100), *hli* (93.8), *trhH* (0), *spvC* (0), *sipA* (0), *sipD* (0), *sopD* (0), *SEN 1417* (0)	
				Kentucky (*n* = 15)	*invA* (100), *mgtC* (100), *sirA* (100), *gipA* (60), *pagK* (93.3), *hli* (80), *trhH* (0), *spvC* (0), *sipA* (0), *sipD* (0), *sopD* (0), *SEN 1417* (0)	
				Anatum (*n* = 5)	*invA* (100), *mgtC* (100), *sirA* (100), *gipA* (40), *pagK* (80), *hli* (0), *trhH* (0), *spvC* (0), *sipA* (0), *sipD* (0), *sopD* (0), *SEN 1417* (0)	
				Infantis (*n* = 3)	*invA* (100), *mgtC* (100), *sirA* (100), *gipA* (0), *pagK* (100), *hli* (0), *trhH* (0), *spvC* (0), *sipA* (0), *sipD* (0), *sopD* (0), *SEN 1417* (0)	
				Muenster (*n* = 3)	*invA* (100), *mgtC* (100), *sirA* (100), *gipA* (33.3), *pagK* (66.7), *hli* (0), *trhH* (0), *spvC* (0), *sipA* (0), *sipD* (0), *sopD* (0), *SEN 1417* (0)	
				Mbandaka (*n* = 2)	*invA* (100), *mgtC* (100), *sirA* (100), *gipA* (100), *pagK* (100), *hli* (0), *trhH* (0), *spvC* (0), *sipA* (0), *sipD* (0), *sopD* (0), *SEN 1417* (0)	

* The percentage (%) of *Salmonella* serotypes is calculated from the positive samples (isolated target bacteria). ND: Not determined.

## Data Availability

No new data were created or analyzed in this study. Data sharing is not applicable to this article.
